# Exploring the Risk Factors for Sudden Infant Deaths and Their Role in Inflammatory Responses to Infection

**DOI:** 10.3389/fimmu.2015.00044

**Published:** 2015-03-05

**Authors:** Caroline Blackwell, Sophia Moscovis, Sharron Hall, Christine Burns, Rodney J. Scott

**Affiliations:** ^1^Faculty of Health and Medicine, Hunter Medical Research Institute, School of Biomedical Sciences, University of Newcastle, Newcastle, NSW, Australia; ^2^Information Based Medicine, Hunter Medical Research Institute, New Lambton, NSW, Australia; ^3^Hunter Area Pathology Service Immunology, John Hunter Hospital, New Lambton, NSW, Australia; ^4^Hunter Area Pathology Service Genetics, John Hunter Hospital, New Lambton, NSW, Australia

**Keywords:** sudden infant death syndrome, inflammation, infection, cigarette smoke, ethnicity

## Abstract

The risk factors for sudden infant death syndrome (SIDS) parallel those associated with susceptibility to or severity of infectious diseases. There is no evidence that a single infectious agent is associated with SIDS; the common thread appears to be induction of inflammatory responses to infections. In this review, interactions between genetic and environmental risk factors for SIDS are assessed in relation to the hypothesis that many infant deaths result from dysregulation of inflammatory responses to “minor” infections. Risk factors are assessed in relation to three important stages of infection: (1) bacterial colonization (frequency or density); (2) induction of temperature-dependent toxins; (3) induction or control of inflammatory responses. In this article, we review the interactions among risk factors for SIDS for their effects on induction or control of inflammatory responses. The risk factors studied are genetic factors (sex, cytokine gene polymorphisms among ethnic groups at high or low risk of SIDS); developmental stage (changes in cortisol and testosterone levels associated with 2- to 4-month age range); environmental factors (virus infection, exposure to cigarette smoke). These interactions help to explain differences in the incidences of SIDS observed between ethnic groups prior to public health campaigns to reduce these infant deaths.

## Introduction

Sudden infant death syndrome (SIDS) is still the major cause of death between 1 month to 1 year of age among infants in industrialized countries. SIDS is a diagnosis of exclusion. The original definition was “… the sudden death of any infant or young child, which is unexpected by history, and in which a thorough post mortem examination fails to demonstrate an adequate cause of death” (1).The definition was revised in 1989 to “the sudden death of an infant under one year of age, which remains unexplained after a thorough case investigation, including performance of a complete autopsy, examination of the death scene, and review of the clinical history” (2) Comparison of epidemiological data from different countries found that infants of some ethnic groups had an increased risk of SIDS (Table 1).

**Table 1:** **Variation in the incidence of SIDS among ethnic groups within countries before the “reduce the risks” campaigns**.

Country	Ethnic group	SIDS/1,000 live births	Reference
Australia	Aboriginal	6.1	(3)
	Non-aboriginal	1.7	
United Kingdom	European	1.7	(4)
	Bangladeshi	0.3	
United States	Total population	2	(5–7)
	Oriental	0.3	
	African-American	5.0	
	Native-American	5.9	
	Alaskan-Natives	6.3	
New Zealand	Maori	7.4	(8, 9)
	Non-Maori	3.6	

The pathogenesis of SIDS has been examined by many disciplines. These have made significant contributions to the study of infant deaths and put forward hypotheses; however, many cannot explain the risk factors or the positive effects of the public health campaigns to reduce the risk (10). The idea that inflammation might be involved in these infant deaths is not new. In an article published in 1956, 126 non-traumatic sudden (“unexplained”) infant deaths were investigated; 106 (84%) revealed microscopic inflammatory changes in 1 or more sites of the respiratory tract, and there was histologic evidence of inflammatory disease in other organs in many cases (11). Table 2 summarizes some of the evidence for inflammatory responses in sudden infant deaths.

**Table 2:** **Inflammatory or immune responses identified in SIDS infants**.

Organ system	Response	Reference
Respiratory tract	Peribronchial inflammatory infiltrates	(12, 13)
	Increase in IgM producing cells in trachea	(14)
	Mast cell degranulation	(15) (Walls, this issue)
Digestive tract	Increased IgA producing cells in duodenum	(14)
	Increased salivary IgA	(16)
Nervous system	Interferon alpha in neurons of the medulla of the brain	(17)
	Increased levels of IL-6 in spinal fluid	(18)
	Lymphocyte infiltration	(19)
Blood	Decreased IgG response to bacterial toxins	(20, 21)
	Increased IgM response to core endotoxin	(21)
	Increased levels of mast cell tryptase	(15)
	Increased levels of mannose binding lectin	(22)
	Cross-linked fibrin degradation products	(23)

## Risk Factors for SIDS and Susceptibility to Infection

Epidemiological studies found significant associations between SIDS and a variety of genetic, developmental, and environmental factors (Table 3). When these factors are compared with those associated with increased susceptibility to bacterial infections, there are remarkable parallels. Each of these factors can affect one or more of the important stages in susceptibility to or severity of infections: frequency or density of bacterial colonization of mucosal surfaces; induction of temperature-dependent toxins; induction or control of inflammatory responses to infection. Each of these is described in detail below.

**Table 3:** **Risk factors for SIDS that parallel risk factors for susceptibility of infants to infection**.

Risks	Reference
**Genetic**
Ethnicity	(3–7, 9, 24)
Male gender	(25–27)
**Developmental**
Night time deaths	(28, 29)
Peak age range 2–4 months	(25)
**Environmental**
Prone sleeping	(25, 30)
Cigarette smoke exposure	(25, 28)
Overheating	(31)
Mild respiratory infections	(25, 31, 32)
Lack of breast feeding	(33)
Poor socio-economic conditions	(25, 34)
No or late immunization	(35, 36)
Air pollution	(37)
Used cot mattress	(38, 39)
Day care	(40)
Older siblings	(41)

Infectious agents or their products have been identified during autopsies of SIDS infants and more recently in sudden unexpected death of infancy (SUDI), which is defined as the sudden and unexpected death of an infant under 1 year of age; SUDI includes explained deaths and those that, after investigation of the death scene and meticulous post-mortem examination, remain unexplained (42). For many years, microbiological findings were dismissed as contamination, overgrowth, or normal flora. More recent studies have questioned these assertions and have provided evidence to support the likelihood that post-mortem bacteriology is a true reflection of infection in these infant deaths (42–44). The common bacterial toxin hypothesis (45–47) considered a number of the risk factors in a mathematical model. It has been supported by identification of potentially toxigenic bacteria or their toxins in SIDS and sudden unexpected deaths in infancy (SUDI) (Table 4). Many of the bacterial toxins or components of the bacteria implicated can act as super antigens, eliciting powerful cytokine storm responses such as those seen in toxic shock syndrome or bacterial sepsis.

**Table 4:** **Toxigenic bacteria and their toxins implicated in sudden death in infancy**.

	Toxin	Reference
*Staphylococcus aureus*	Enterotoxins, TSST^a^	(48–50)
*Bordetella pertussis*	Pertussis toxin, endotoxin^a^	(51–53)
*Haemophilus influenzae*	Endotoxin^a^	(54)
*Clostridium perfringens*	Enterotoxin A^a^	(55, 56)
*Clostridium botulinum*	Botulism toxin	(57–59)
*Streptococcus pyogenes*	Pyrogenic toxins A and B^a^	(54)
*Escherichia coli*	Enterotoxins, verotoxins	(60–63)
	Curlin^a^	(64)
*Helicobacter pylori*	Endotoxin, vacuolating^a^ toxin, urease	(65)
*Pneumocystis*	?	(66)

*^a^Superantigenic activity*.

*?, toxin/antigen unknown*.

The infection hypothesis does not fit Koch’s postulates. That is, there is no single organism implicated and there is no widely accepted animal model that reflects the genetic, developmental, and environmental risk factors identified in epidemiological studies (see Blood-Siegfried, this issue). In addition, a variety of viruses have been identified in SIDS infants (67–69). Sterile site infections have been identified in SUDI, and a variety of toxigenic bacteria or their toxins have been reported in these infant deaths (Table 4). Our hypothesis is that there is not a particular organism or toxin, the factor in these deaths is the dysregulation of the inflammatory responses elicited by what appear to be mild or asymptomatic infections.

The objective of this review was to assess how the risk factors identified in epidemiological studies of SIDS affect susceptibility to infection and/or alter inflammatory responses to infections. It addresses the interactions between these identified risk factors and the three key stages of infection: (1) increased frequency or density of bacterial colonization; (2) induction of temperature-dependent toxins; (3) induction or control of inflammatory responses (Table 5).

**Table 5:** **How do risk factors for SIDS affect susceptibility to infection**.

Effects on frequency or density of colonization by bacteria by
Developmental stage/expression of receptors
Prone position
Virus infection
Cigarette smoke
Day care
Older siblings
Effects on temperature-dependent bacterial toxin production by
Overheating
Prone position
Virus infection
Effects on induction or control of inflammatory responses by
Cigarette smoke
Virus infection
Sex
Genetic background
Developmental stage (low maternal antibodies, cortisol levels, testosterone surge)

## Bacterial Colonization

Factors affecting colonization have been elucidated in previous studies. Virus infections, which often precede SIDS or SUDI, can enhance bacterial binding through induction of host receptors for bacteria or induction of new receptors (70, 71). The prone position can lead to pooling of respiratory secretions and increased numbers and varieties of bacteria, particularly in the presence of virus infection (72). Active smoking can predispose individuals to virus infections, and smokers are colonized more heavily and more frequently with potential pathogens (73). In addition to enhancing susceptibility to virus infections, material in cigarette smoke can passively coat epithelial cells and enhance “stickiness” for potential pathogens (74). Exposure of infants to new infectious agents can be enhanced by day care with other children or older siblings attending nursery or school outside the home environment.

## Temperature-Dependent Toxins

The pyrogenic toxins of *Staphylococcus aureus* have been identified in over half of SIDS infants from five different countries (50). *S. aureus* is the most common isolate from the nasopharynx of healthy infants, and 64% of these have the capacity to produce these toxins (75). The toxins are induced only between 37 and 40°C, and the temperature of the nasopharynx is usually below this range (76). In the prone position, the temperature in the nasopharynx of children is increased and 15% had temperatures ≥37°C. While the toxigenic organisms are present in most infants, induction of the toxin is likely to be dependent on risk factors such as overheating, prone position, or virus infection with associated blocked nasal passages that result in reduced cooling effects of the passage of air.

## Inflammation and SIDS

The common thread in these deaths is not a single organism or toxin; it is dysregulation of the innate inflammatory responses in the non-immune infant or young child who encounters a new potential pathogen. There is recent evidence that a balanced inflammatory response during infancy is important for survival. In a study of low birth weight infants, cytokine responses were assessed *in vitro* by stimulation of whole blood cultures with lipopolysaccharide (LPS), phytohemagglutinin (PHA), or purified protein derivative (PPD). Infants whose cytokine responses to LPS or PHA were very low or very high were at increased risk of death assessed by survival of the cohort tested. The interpretation of these findings was that a balanced response to new pathogens was more likely to result in survival (77). A variety of bacteria implicated in SIDS/SUDI possess structural antigens or exotoxins that can act as superantigens (e.g., LPS) that can activate large populations of inflammatory cells (Table 3).

Inflammation can be assessed from clinical evidence, histological findings, or molecular markers. Clinical evidence of inflammation has been identified in some of these infant deaths. Infants were very hot when found and remained hot for several hours after death (78). Histological signs of inflammation are considered the gold standard by the clinical pathologist and some of these are summarized in Table 2. It has been suggested that molecular markers of inflammation need to be measured; however, these are relatively expensive and are not usually included in standard autopsy protocols. To date, there have been few studies of inflammatory markers in sudden deaths (18, 79, 80). Because these components are not regularly examined during autopsy, co-operation with a research group with an interest in inflammation would help to obtain evidence for these markers.

## Inflammation in Relation to Proposed Mechanisms of Death in SIDS

**Figure 1:**
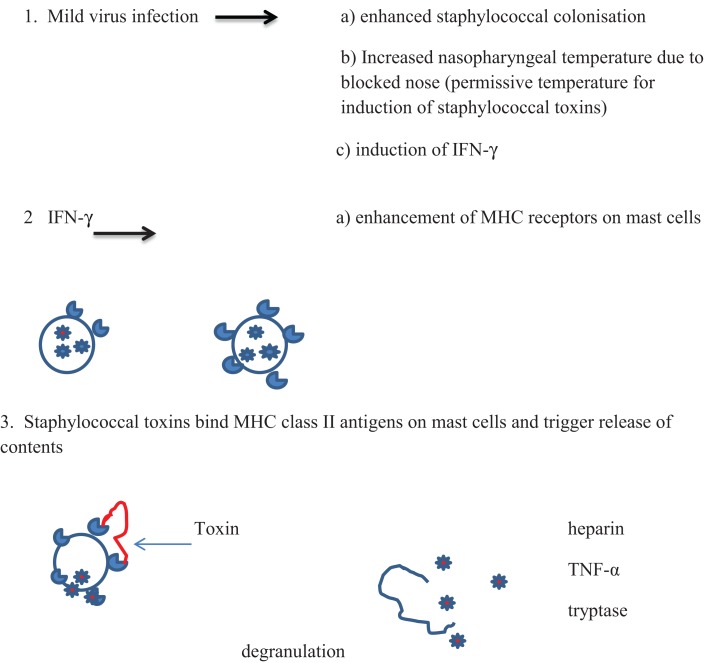
**Proposed scheme for interactions between virus infections and pyrogenic staphylococcal toxins in non-antibody-mediated induction of mast cell degranulation (see text)**.

Cytokines produced in the inflammatory responses to infectious agents could have an impact on most of the mechanisms of death proposed: anaphylaxis; poor arousal; hypoxia and apnea; shock; cardiac arrythmias; hyperthermia; hypoglycemia (81). Anaphylaxis was the first mechanism proposed in an animal model in which guinea pigs were sensitized to cow’s milk (82, 83). Studies of SIDS infants have not found evidence to support the hypothesis that IgE-mediated anaphylaxis is involved in these infant deaths. There is evidence, however, for degranulation of mast cells in some infants (15) (Walls, this issue). In addition, in an *in vitro* study of first degree relatives of SIDS infants or infants who had suffered an acute life threatening episode (ALTE), there was evidence of increased mast cell hyper-releasability and degranulation (84). This could be mediated by some of the pyrogenic staphylococcal toxins through non-antibody activation of mast cells. The toxins, which are super antigens, can bind directly to the Vβ antigens on mast cells and trigger degranulation independent of antibody and complement. A mild virus infection might enhance colonization by *S. aureus* and increased temperature of the oropharynx due to blocked nasal passages. This could result in temperatures that are permissive for induction of the staphylococcal toxins. Increased production of interferon-γ (IFN-γ) and/or other mediators could enhance expression of Vβ antigens which are receptors for the toxins. Cross-linking of the Vβ antigens by the toxins could lead to non-IgE-mediated degranulation of mast cells (Figure 1). Evidence of mast cell tryptase and other products of mast cells have been published (15) (Walls, this volume).

## *In vitro* Studies of Inflammation in Human Material

The inflammatory responses can be affected by genetic, developmental, and environmental factors. *In vitro* studies need to consider these potential confounding factors. Taking blood from young infants is not practical for most studies, so cell lines or adult leukocytes have been used to assess the effects of risk factors for SIDS on inflammation. In addition to controlling for confounding factors, it is important to use biologically relevant concentrations of substances to be assessed in the model system (85).

While bacteria and their toxins have been implicated in SIDS, there is epidemiological and experimental evidence for virus infections acting as a cofactor. Many parents reported their child had a mild cold or sniffle prior to death. The presence of a risk factor such as prone sleeping, head covered, or parental smoking combined with infection was associated with a greater risk of SIDS than the individual risk factor alone (32).

In animal models, the lethal doses of both staphylococcal exotoxins (86) and endotoxin (87–89) were significantly reduced if the animals had mild/asymptomatic virus infection. Virus infections induce IFN-γ, which can significantly enhance production of pro-inflammatory cytokines (Moscovis et al., this volume). IL-6 has been identified in the CSF of SIDS infants (18). We demonstrated a dose-dependent enhancement by IFN-γ of IL-6 elicited by LPS in a human cell line and human peripheral blood monocytic cells (PBMC) (85). In addition, in a model system, IFN-γ has been demonstrated to reduce significantly the anti-inflammatory IL-10 responses elicited from human PBMC exposed to endotoxin (Moscovis et al., this volume).

In contrast, components of cigarette smoke, such as nicotine (or its liver metabolite cotinine), can suppress endotoxin-induced IFN-γ, IL-1β, TNF-α, and IL-8. There are, however, limitations in using single purified reagents for *in vitro* studies as the responses elicited might not reflect accurately the complex interactions that occur *in vivo*. Nicotine is only one of approximately 4,000 chemicals in cigarette smoke. A water soluble cigarette smoke extract (CSE) enhanced IL-8 responses but reduced other pro-inflammatory cytokines (85). *In vitro* studies found cells from smokers produced significantly lower anti-inflammatory IL-10 responses than cells of non-smokers (90). This could impair the ability to control pro-inflammatory responses to infection.

## Developmental Stage and SIDS

The mathematical model used for the common bacterial toxin hypothesis predicted the 2- to 4-month age range as that during which the peak of SIDS occurred. This prediction fits the epidemiological data. There are a number of factors that could contribute to susceptibility to infection during his period: (1) loss of maternal antibody; (2) development of circadian rhythms and the associated changes in night time cortisol; (3) the testosterone surge in males between 1 and 5 months.

### Antibody levels and the effects of immunization

An important factor is the loss of maternal antibodies, which make the infants more reliant on their innate inflammatory responses to cope with new infections. This can include exposure to new infections brought home by older siblings or attendance at day care, both of which have been identified as risk factors for SIDS in epidemiological studies. Immunization has been demonstrated to be a protective factor in relation to SIDS (35). Most infants start their immunizations during the period of peak vulnerability, but they will not be fully immunized to common childhood illnesses implicated in some epidemiological studies (e.g., whooping cough) by 4 months of age. The protective effect of immunization was noted in early studies (35), and a shift in the age range of SIDS in infants in both Scotland and England was observed following changes in the immunization of infants in UK. That is, immunization began at 2 months instead of 3 months (91). It has also been demonstrated that some cross protection against staphylococcal exotoxins was elicited in animals by tetanus toxoid (91).

### Development of circadian rhythm and hormonal changes

**Figure 2:**
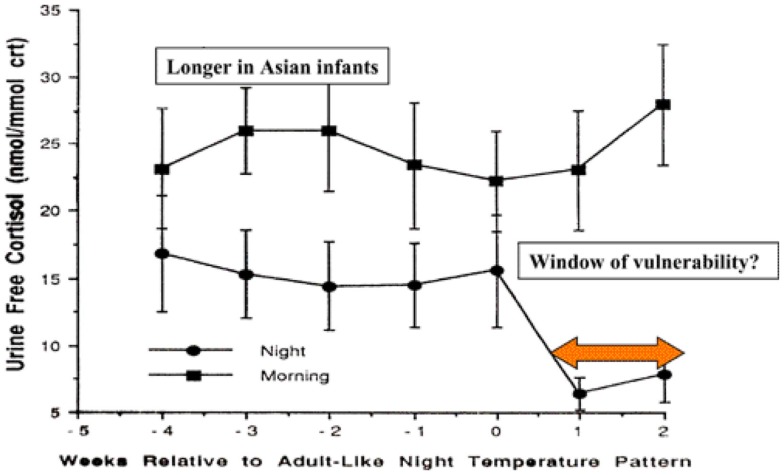
**Day time and night time cortisol levels of infants in relation to development of circadian rhythm and potential for control of inflammatory responses to infection [adapted from Ref. (92)]**.

During the first months of life, significant physiological changes occur which could affect control of the inflammatory responses. Circadian rhythm develops between 4 and 16 weeks in Caucasian infants in Britain, but this does not occur until 12–20 weeks in Asian infants (92). The switch to circadian rhythm is measured by the night time drop in core body temperature. This physiological switch is accompanied by a dramatic drop in night time, but not daytime, cortisol levels (Figure 2).

The levels of night time cortisol after the switch were not capable of reducing pro-inflammatory responses in a model system examining inflammatory responses of peripheral blood monocytes to staphylococcal toxins (93). Daytime levels and night time levels prior to the switch were able to reduce these responses. If these *in vitro* studies reflect the activity of cortisol *in vivo* in the infant, this 2- to 4-month period could be a window of vulnerability to new infections, particularly at night. The circadian switch occurs later among Asian infants (12–20 weeks), which allows time for natural exposure to infection or immunization to boost specific immunity. Prior to the public health campaigns to reduce the risks of SIDS, there was a lower incidence of SIDS among Asian infants in UK compared with infants of European origin (4).

### The effect of testosterone

The male excess in SIDS noted in epidemiological studies and assessed in mathematical models (26) might reflect hormonal changes that occur during the period in which SIDS is most prevalent. There is a rise in testosterone production associated with the period during which SIDS is most prevalent. Between 1 and 5 months, testosterone levels range from 0.03 to 6.14 nMol L^−1^ for males and 0.03 to 0.17 nMol L^−1^ for females. In males, these levels decrease to 0.07–0.24 at 6–11 months (94). In a previous study (95), the ranges of testosterone in the adult females (<0.4–3.1 nMol L^−1^) tested were within the range for males in the 1- to 5-month age range (94). In the model system examined, there was a positive correlation between testosterone levels and pro-inflammatory responses to LPS when the cells were pre-treated with IFN-γ or IFN-γ and CSE. No correlations were observed for the higher levels (6.3–21 nMol L^−1^) found in adult males (95).

These findings indicate that in addition to low levels of night time cortisol present in infants over the age range of greatest risk of SIDS (93), dysregulation of the inflammatory responses to apparently “mild” infections might be amplified by the increase in testosterone in male infants at this time. As this does not occur in female infants, this could be an additional factor contributing to the higher proportion of males among SIDS infants. *In vitro* studies using the PBMC model might provide additional insights into the interactions between cortisol and testosterone levels noted in infants during this critical age range.

### Interactions with genetic factors

Among the ethnic groups at increased risk of SIDS (e.g., indigenous populations in Australia and North America and Black Americans), there is a higher proportion of genotypes associated with strong pro-inflammatory responses (96–98). While these disparities have been ascribed primarily to socio-economic disadvantage, there is emerging evidence that genetic background and interactions between environmental factors such as cigarette smoke might contribute to susceptibility and severity of infections (85, 90). Of particular importance is the effect of cigarette smoke on the genotype of IL-10 (G-1082A) associated with lower levels of the anti-inflammatory response. The AA genotype is predominant among the groups at higher risk of SIDS, and in our *in vitro* studies, cells from smokers with this genotype had the lowest responses (90). Among the ethnic groups at increased risk of SIDS, there is also a higher incidence of maternal smoking. South Asians have genetic profiles similar to those for the higher risk groups; however, smoking is much less prevalent among south Asian women in UK.

Our experimental studies indicate that higher IFN-γ responses might elicit higher pro-inflammatory responses to bacterial toxins (Moscovis et al., this volume). Indigenous Australians tested had the highest proportion of individuals with the TT genotype of *IFNG* T + 874A (96), which is associated with high IFN-γ responses observed *in vitro* (99).

## Conclusion

There is a wealth of knowledge about the risk factors for sudden death in infancy; it is important, however, to attempt to explain how these risk factors could result in death. There is a growing body of evidence that infection and inflammatory responses might trigger the events leading to sudden death in infancy. A recent review on infectious causes of SIDS concluded no specific organism was involved in SIDS (100). The evidence of Anderson et al. (77) indicates that a balanced inflammatory response is important for dealing with new infections. Our hypothesis is that the common thread in these deaths is dysregulation of the inflammatory responses to apparently mild infections. The risk factors identified in epidemiological studies can have significant effects on inflammatory responses that could affect the different physiological mechanisms proposed to explain these infant deaths (81). Many of the genetic, developmental, and environmental factors identified could affect this balance resulting in enhanced pro-inflammatory cytokine levels, which can affect glucose levels, heart rate, apnea, arousal, anaphylaxis, and shock. The components of the inflammatory responses involved might differ in individual children; however, it is the dysregulation of the responses that lead to the death of the child. We need to investigate further the interactions between genetic, developmental, and environmental risk factors on inflammatory responses to attempt to identify infants at increased risk and to attempt to introduce measures to prevent induction of these lethal responses.

## Author Contributions

Each of the authors made substantial contributions to the conception, design, analyses, and interpretations of the work. They assisted in preparing the article, critically assessed the final version, and agree to be accountable for the accuracy and integrity of the work.

## Conflict of Interest Statement

The authors declare that the research was conducted in the absence of any commercial or financial relationships that could be construed as a potential conflict of interest.
